# Economic benefits of sharing and redistributing influenza vaccines when shortages occurred

**DOI:** 10.1371/journal.pone.0186418

**Published:** 2017-10-17

**Authors:** Sheng-I Chen

**Affiliations:** Department of Industrial Engineering and Management, School of Management, National Chiao-Tung University, Hsinchu, Taiwan; Centers for Disease Control, TAIWAN

## Abstract

**Background:**

Recurrent influenza outbreak has been a concern for government health institutions in Taiwan. Over 10% of the population is infected by influenza viruses every year, and the infection has caused losses to both health and the economy. Approximately three million free vaccine doses are ordered and administered to high-risk populations at the beginning of flu season to control the disease. The government recommends sharing and redistributing vaccine inventories when shortages occur. While this policy intends to increase inventory flexibility, and has been proven as widely valuable, its impact on vaccine availability has not been previously reported.

**Material and methods:**

This study developed an inventory model adapted to vaccination protocols to evaluate government recommended polices under different levels of vaccine production. Demands were uncertain and stratified by ages and locations according to the demographic data in Taiwan.

**Results:**

When vaccine supply is sufficient, sharing pediatric vaccine reduced vaccine unavailability by 43% and overstock by 54%, and sharing adult vaccine reduced vaccine unavailability by 9% and overstock by 15%. Redistributing vaccines obtained greater gains for both pediatrics and adults (by 75%). When the vaccine supply is in short, only sharing pediatric vaccine yielded a 48% reduction of unused inventory, while other polices do not improve performances.

**Conclusions:**

When implementing vaccination activities for seasonal influenza intervention, it is important to consider mismatches of demand and vaccine inventory. Our model confirmed that sharing and redistributing vaccines can substantially increase availability and reduce unused vaccines.

## Introduction

Seasonal influenza has been a concern in Taiwan, as more than three million patients visit clinics each year due to influenza-like infections. Of these, the infected population accounts for 45% of overall children under 4 years old, 22% of all aged 5 to 14, 7% of all aged 15 to 24, 8% of all aged 25 to 64, and 18% of all aged 65 years or older [1]. During the 2013–2014 season, close to 2,000 severe complications were reported among these infected individuals, and 151 mortalities were found to be associated with influenza infections [2].

**Fig 1:**
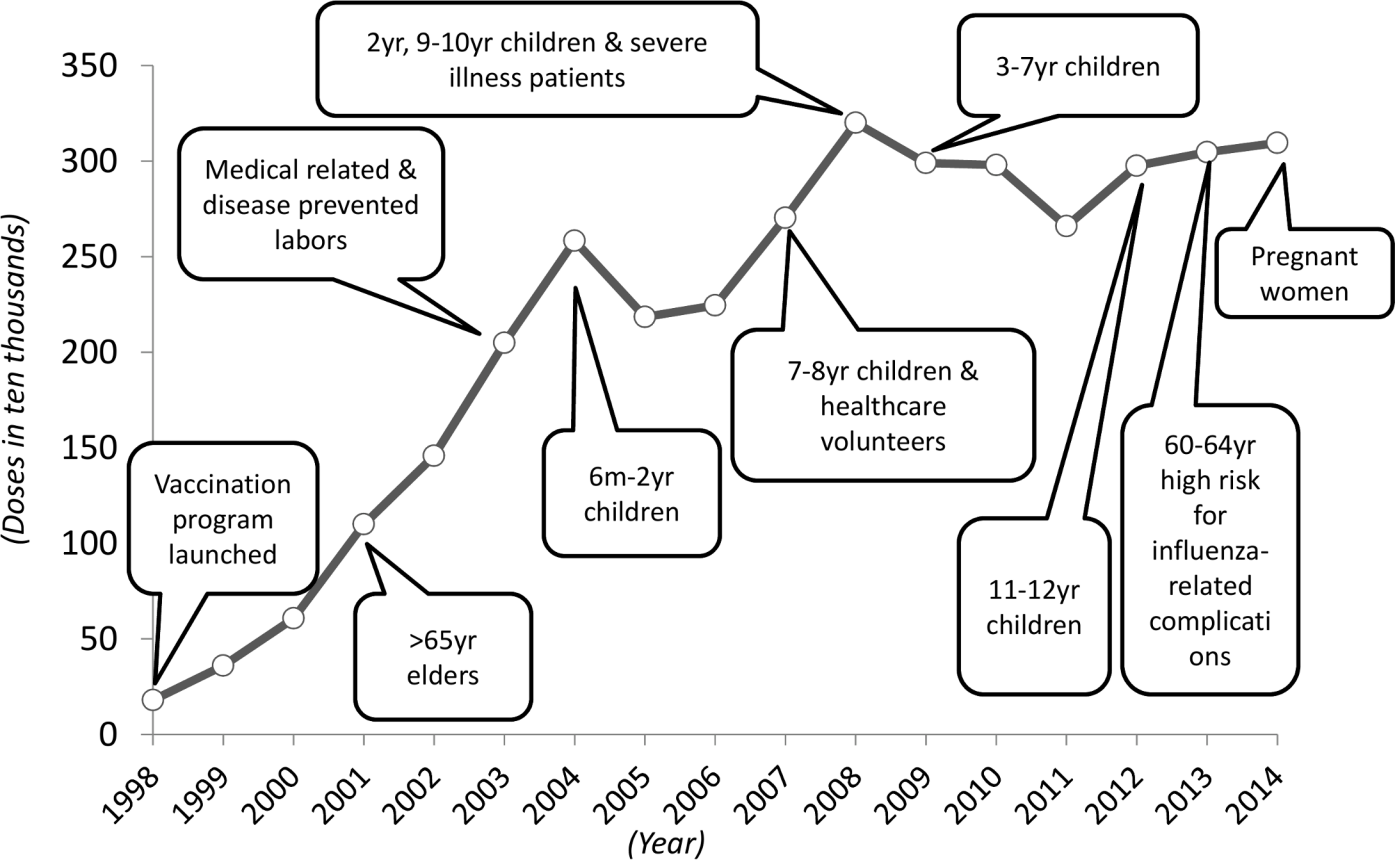
Vaccination doses and coverage populations in Taiwan.

Vaccination is a common approach to protect people from infection [3,4]. Many studies demonstrate the benefits of using vaccines against seasonal influenza [5–7]. Specific papers emphasize on the effectiveness of influenza intervention with the consideration of the population in Taiwan [8–12]. An emerging research direction has utilized economic analysis to assess the he economic benefits from both health service, societal, and other perspectives [13–16]. The first vaccination program in Taiwan was launched in 1998. The population aged 65 and above, those aged 60 to 64 and at high risk of influenza-related complications, healthcare workers and volunteers, children aged 6 months to 12 years, and pregnant women were all covered by the program by 2014, with approximately three million doses administered annually [17–19]. Fig 1 summarizes vaccination program reforms for the covered populations and vaccine doses.

Two types of vaccines are currently ordered by the government to control seasonal influenza: 0.25ml and 0.5ml per dose trivalent vaccines. The first vaccine type is primarily applied in children less than three years old, and the second is allocated to the other age groups in the population. Despite the government supporting free vaccines, vaccination rates remain low in most high-risk population groups in Taiwan [20–22]. Only approximately 35% of the elderly population (65 years and above) were administered government-funded vaccines, and the vaccination rate is much lower than the threshold necessary to establish herd immunity [23].

Demand forecasting is the first operation in preparing national immunization programs. Each county estimates its vaccine demands according to the previous season’s vaccination rates and target populations [24]. The Centers for Disease Control (CDC) then consolidates each county demand, and places orders to vaccine manufacturers [18]. Annual vaccinations start on October 1 at thousands of certificated health institutes nationwide [25]. As forecast errors can lead to imbalances between vaccine demand and supply, the government recommends sharing and redistributing inventories when a shortage occurs for any vaccine product at any location [26]. These policies attempt to mitigate forecast errors by aggregating multiple vaccine inventory sources. Although inventory pooling has been noted as valuable to broaden areas, its application in vaccinations is rarely examined. This study aims to investigate this unexplored application by assessing inventory performances of various policies.

## Methods

This study develops stochastic inventory models for assessing the economic benefits of sharing and redistributing vaccines. The population is divided into two age groups and each one uses a specific vaccine product. Sharing vaccine with other age group is possible when the matched vaccine inventory is in short. The first subsection describes detail operations and dose usages of these sharing practices. Vaccine demands at each county are based on the actual population distribution and arrival rates in Taiwan. The stochastic inventory models are then constructed to obtain the expected vaccine unavailability and overstocks under the following four policies: no sharing and redistributing vaccines, sharing vaccines only, redistributing vaccines only, combination of sharing and redistributing vaccines.

### Sharing and redistributing vaccines

**Fig 2:**
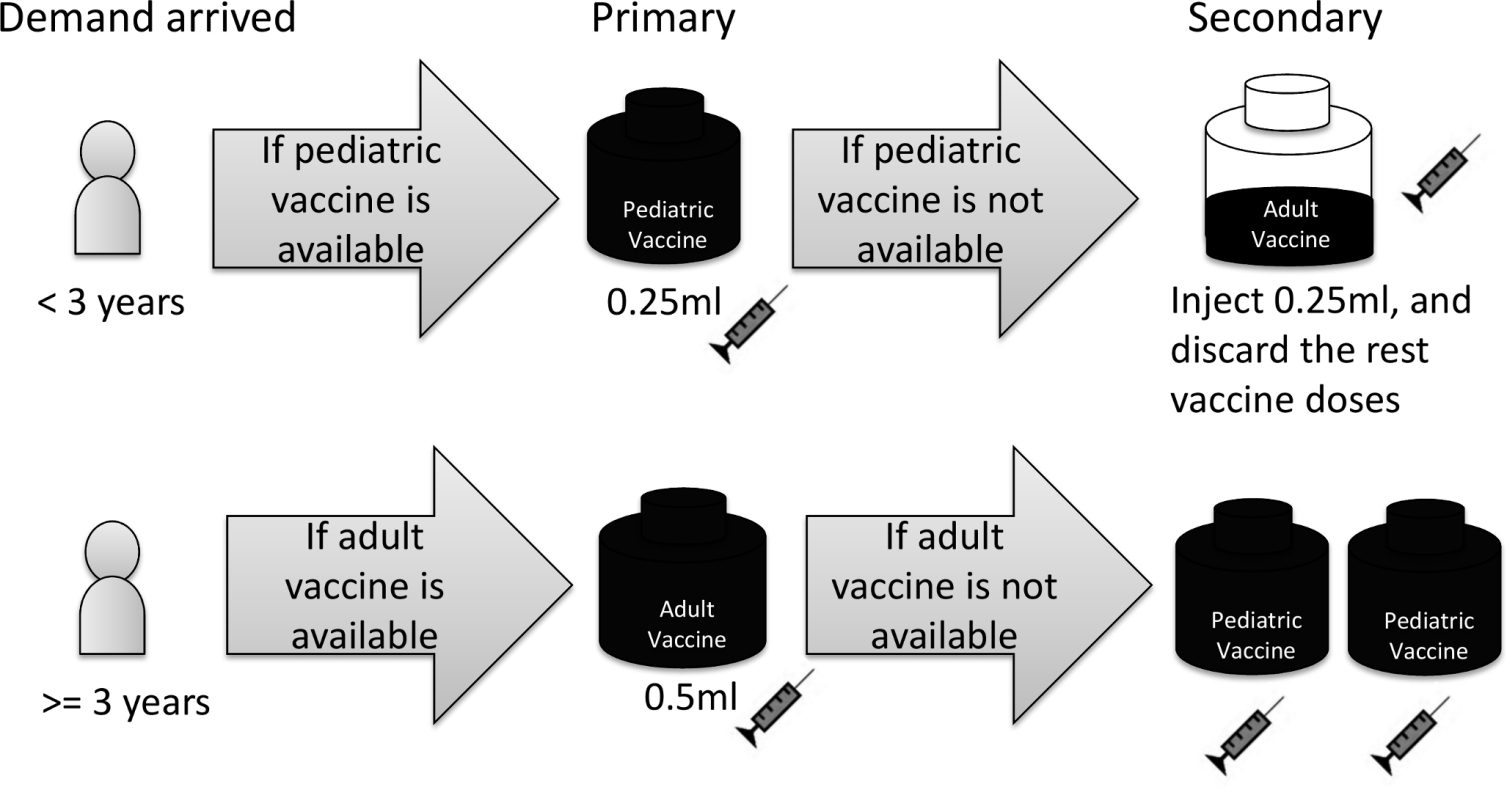
The schematic of sharing pediatric and adult influenza vaccines.

Vaccines are redistributed across different counties when inventory is depleted during the season. We model such a policy by assuming that inventories are held at a centralized warehouse to supply nationwide demand. Another policy we studied is the sharing of pediatric and adult vaccines by all age group populations. As recommended by the Taiwanese government, individuals may use substitute vaccines as an alternative to develop immunizations against influenza. With this scheme, children younger than three years old are vaccinated using a half vial of adult vaccine when pediatric vaccine is in short supply. The remaining dose in the opened vial should be discarded, and it cannot be used for any other recipients [26]. In another situation, when there are extra pediatric vaccines at the end of vaccination season. The United States’ CDC recommends individuals aged three years and older may take two doses of pediatric vaccine as an alternative to the adult vaccines [27]. Fig 2 is used to summarize the vaccine-sharing schemes based on this information:

### Population data

The population data is collected from the Department of Statistics in the year of 2014, in which New Taipei City has the most residences, followed by Taipei City, Kaohsiung City, Taichung City, Taoyuan City, and so on [28]. The target population included children 6 months to 12 years and population 65 years and above. We approximated children between 6 months to 1 year by using the reported numbers of children aged between 0 to 12 months divided by 2. Each county’s population is further stratified by 6 months ~ 3 and ≥ 3 years, representing child and adult vaccine demands, respectively. Table 1 displays target population distributions.

**Table 1:** Target population distributions.

County	All Age Groups		6 months ~ 3 Years		≥ 3 Years	
	Populations	Percentages	Populations	Percentages	Populations	Percentages
New Taipei City	822,481	15.23%	90,152	1.67%	732,329	13.56%
Taipei City	684,961	12.68%	76,585	1.42%	608,376	11.27%
Kaohsiung City	616,720	11.42%	57,521	1.07%	559,199	10.35%
Taichung City	595,688	11.03%	66,586	1.23%	529,102	9.80%
Taoyuan County	450,607	8.34%	46,817	0.87%	403,790	7.48%
Tainan City	433,601	8.03%	40,372	0.75%	393,229	7.28%
Changhua County	317,474	5.88%	29,392	0.54%	288,082	5.33%
Pingtung County	198,714	3.68%	14,251	0.26%	184,463	3.42%
Yunlin County	186,929	3.46%	13,529	0.25%	173,400	3.21%
Miaoli County	144,430	2.67%	14,608	0.27%	129,822	2.40%
Hsinchu County	136,112	2.52%	15,390	0.28%	120,722	2.24%
Chiayi County	135,304	2.51%	8,333	0.15%	126,971	2.35%
Nantou County	126,317	2.34%	9,221	0.17%	117,096	2.17%
Yilan County	111,191	2.06%	9,054	0.17%	102,137	1.89%
Hsinchu City	106,962	1.98%	13,652	0.25%	93,310	1.73%
Keelung City	80,281	1.49%	5,497	0.10%	74,784	1.38%
Hualien County	79,843	1.48%	6,883	0.13%	72,960	1.35%
Chiayi City	65,486	1.21%	5,246	0.10%	60,240	1.12%
Taitung County	55,148	1.02%	4,481	0.08%	50,667	0.94%
Kinmen County	25,357	0.47%	3,270	0.06%	22,087	0.41%
Penghu County	24,213	0.45%	2,312	0.04%	21,901	0.41%
Lienchiang County	2,477	0.05%	376	0.01%	2,101	0.04%
Total	5,400,292	100.00%	533,524	9.88%	4,866,768	90.12%

### Vaccination rates

Actual vaccination rates vary across time, but no such data has been reported every year, to the best of our knowledge. We use the most recent data available from the Taiwanese CDC for the 2011–2012 influenza season [29]. The first-time recipients with 6 months through pre-school age are required to receive the second dose. Children aged 6 months through 1 year are all considered as the first time recipients, and the percentage of first-time recipients of other age group children are approximated according to the reported numbers of partial and complete vaccination recipients.

### Inventory models

We assume that pediatric and adult vaccine demands at the 22 locations are uncertain and distributed heterogeneously. Their means are determined by the target population and the vaccination rate. The index of vaccine type is denoted as *i*, where *i = 1* represents the pediatric vaccine, and *i = 2* represents the adult vaccine. The index *j* represents to the location. Thus, the notation *q*_*i*,*j*_ is the ordering quantity of vaccine *i* at location *j*. ***X***_***i*,*j***_ is a random demand of vaccine type *i* at location *j* with a mass function *p*_*i*,*j*_ (.). In the case study, we assume that pediatric and adult vaccine demands at each location are Poisson distributed with a mean equal to the age group population multiplied by the vaccination rate. However, our model is capable to adopt with general distributions. The expected overstocks and unavailability are denoted as *w*_*i*,*j*_ and *π*_*i*,*j*_, respectively.

#### Policy 1. No sharing and redistributing vaccines (baseline)

We first formulate the baseline policy by assuming that vaccines are stocked at the 22 counties separately. Further, both pediatric and adult vaccines can only apply to specific age group populations. The expected unavailability of vaccine type *i* at location *j* can be expressed as:

$$\pi_{i,j}(q_{i,j})={E[X_{i,j}-q_{i,j}]}^{+}$$ (1)

and the expected overstock of vaccine type *i* at location *j* is as follows:

$$w_{i,j}(q_{i,j})={E[q_{i,j}-X_{i,j}]}^{+}.$$ (2)

### Policy 2. Sharing vaccines

Next, we formulate the policy for sharing pediatric and adult vaccines. Suppose that both vaccine types can be administered to all age group populations when one of the vaccine types is out of stock. As aforementioned, a child younger than 3 years old uses half-doses of an adult vaccine, and the remaining half doses are discarded. Another substitution scheme, each adult receives two doses of pediatric vaccine. Given the ordering quantities of pediatric and adult vaccines *q*_1,*j*_ and *q*_2,*j*_, the expected unavailability for pediatrics is:

$$\begin{array}{l} \pi_{1,j}(q_{1,j},\;q_{2,j})=\sum_{n_{2}=q_{2,j}}^{\infty }\sum_{n_{1}=q_{1,j}}^{\infty }\;\left(n_{1}-q_{1,j})p_{1,j}(n_{1})\;p_{2,j}(n_{2}\right)\\ +\sum_{n_{2}=0}^{q_{2,j}}\sum_{n_{1}=q_{1,j}+q_{2,j}-n_{2}}^{\infty }\;\left(n_{1}-q_{1,j}-(q_{2,j}-n_{2}))p_{1,j}(n_{1})\;p_{2,j}(n_{2}\right). \end{array}$$ (3)

The expected value of Eq (3) depends on the inventory levels of both pediatric and adult vaccines. The first term at the right-hand side computes the expected shortages when adult vaccines are out of stock (i.e. *n*_2_ ≥ *q*_2,*j*_). The second term captures the situation of using leftover adult vaccines to cover pediatric demands. For example, when an adult vaccine has one dose left over (i.e., *q*_2,*j*_ – *n*_2_ = 1) and unmet pediatric demand equals 1 (i.e. *n*_1_ − *q*_1,*j*_ = 1), the extra adult vaccine can be used through excess pediatric demand, and therefore, no shortage cost has occurred.

The formulation of expected unavailability for adults is similar to the pediatrics. The only difference is that each excess adult demand requires two doses of pediatric vaccine (i.e., $\left\lfloor \frac{q_{1,j}-n_{1}}{2}\right\rfloor$). The expected unavailability of adults at location *j* is:

$$\begin{array}{l} \pi_{2,j}(q_{1,j},\;q_{2,j})=\sum_{n_{2}=q_{2,j}}^{\infty }\sum_{n_{1}=q_{1,j}}^{\infty }\;\left(n_{2}-q_{2,j})p_{1,j}(n_{1})\;p_{2,j}(n_{2}\right)\\ +\sum_{n_{1}=0}^{q_{1,j}}\sum_{n_{2}={q_{2}+\lfloor \frac{q_{1,j}-n_{1}}{2}\rfloor }_{,j}}^{\infty }\;\left(n_{2}-q_{2,j}-\lfloor \frac{q_{1,j}-n_{1}}{2}\rfloor )p_{1,j}(n_{1})\;p_{2,j}(n_{2}\right). \end{array}$$ (4)

Eq (5) represents the expected overstock of pediatric and adult vaccines. We first consider a situation in which pediatric vaccines are left over at the end of the season, even after the inventories are partially consumed by adult demands. The first term captures the case in which both the remaining pediatric and adult vaccines are unused. The second term considers a situation in which there are extra pediatric vaccines while the adult vaccines run out. The expected overstock of pediatric vaccines at location *j* is the following:

$$\begin{array}{l} w_{1,j}(q_{1,j},\;q_{2,j})=\sum_{n_{2}=0}^{q_{2,j}}\sum_{n_{1}=0}^{q_{1,j}}\;(q_{1,j}-n_{1})p_{1,j}(n_{1})\;p_{2,j}(n_{2})\\ +\sum_{n_{2}=q_{2,j}}^{\infty }\sum_{n_{1}=0}^{q_{1,j}-2(n_{2}-q_{2,j})}\;(q_{1,j}-n_{1}-2(n_{2}-q_{2,j}))p_{1,j}(n_{1})\;p_{2,j}(n_{2}). \end{array}$$ (5)

The expected overstock of adult vaccines at location *j* is formulated as Eq (6). The first term represents the overstock of adult vaccines when both vaccines types have excess inventories at the end of the season. The second term computes the expected overstocks in the situation when adult vaccines have remaining doses and pediatric vaccines are depleted.

$$\begin{array}{l} w_{2,j}(q_{1,j},{\;q}_{2,j})=\sum_{n_{1}=0}^{q_{1,j}}\sum_{n_{2}=0}^{q_{2,j}}\;(q_{2,j}-n_{2})p_{1,j}(n_{1})\;p_{2,j}(n_{2})\\ +\sum_{n_{1}=q_{1,j}}^{\infty }\sum_{n_{2}=0}^{q_{2,j}-(n_{1}-q_{1,j})}\;(q_{2,j}-n_{2}-(n_{1}-q_{1,j}))p_{1,j}(n_{1})\;p_{2,j}(n_{2}). \end{array}$$ (6)

### Policy 3. Redistributing vaccines

This policy allows vaccine inventories to be redistributed within different locations, yet pediatric and adult vaccines are not shared among both age group populations. Thus, the expected unavailability of vaccine type *i* is:

$$\pi_{i}(q_{i})={E[X_{i}-q_{i}]}^{+},$$ (7)

and the expected overstock of vaccine type *i* is the following:

$$w_{i}(q_{i})={\;E[q_{i}-X_{i}]}^{+}$$ (8)

Note that Eqs (7) and (8) are similar to Eqs (1) and (2), except Eqs (7) and (8) do not include the subscript *j*. Another implication of this policy may refer to the centralized inventory system, where vaccines are kept in a warehouse and then delivered to demand sites as requested. To compute the expected value, we aggregate the demand data and ordering quantities from the 22 locations in nationwide.

### Policy 4. Combination of sharing and redistributing vaccines

This policy allows demands to be fulfilled by inventories, regardless of vaccine types and locations. By combining Policies 2 and 3, we formulate the expected unavailability of pediatric vaccines as:

$$\begin{array}{l} \pi_{1}(q_{1},{\;q}_{2})=\sum_{n_{2}=q_{2,j}}^{\infty }\sum_{n_{1}=q_{1}}^{\infty }\left(n_{1}-q_{1})p_{1}(n_{1})\;p_{2}(n_{2}\right)\\ +\sum_{n_{2}=0}^{q_{2}}\sum_{n_{1}=q_{1}+q_{2}-n_{2}}^{\infty }\;(n_{1}-q_{1}-(q_{2}-n_{2}))p_{1}(n_{1})\;p_{2}(n_{2}), \end{array}$$ (9)

the expected unavailability of adult vaccines as:

$$\begin{array}{l} \pi_{2}(q_{1,j},{\;q}_{2,j})=\sum_{n_{2}=q_{2}}^{\infty }\sum_{n_{1}=q_{1}}^{\infty }\left(n_{2}-q_{2})p_{1}(n_{1})\;p_{2}(n_{2}\right)\\ +\sum_{n_{1}=0}^{q_{1}}\sum_{n_{2}=q_{2}+\lfloor \frac{q_{1}-n_{1}}{2}\rfloor }^{\infty }\left(n_{2}-q_{2}-\lfloor \frac{q_{1}-n_{1}}{2}\rfloor )p_{1}(n_{1})\;p_{2}(n_{2}\right), \end{array}$$ (10)

the expected overstock of pediatric vaccines as:

$$\begin{array}{l} w_{1}(q_{1},{\;q}_{2})=\sum_{n_{2}=0}^{q_{2,j}}\sum_{n_{1}=0}^{q_{1,j}}\;(q_{1}-n_{1})p_{1,j}(n_{1})\;p_{2}(n_{2})\\ +\sum_{n_{2}=q_{2}}^{\infty }\sum_{n_{1}=0}^{q_{1}-2(n_{2}-q_{2})}\;(q_{1}-n_{1}-2(n_{2}-q_{2}))p_{1}(n_{1})\;p_{2}(n_{2}), \end{array}$$ (11)

and the expected overstock of adult vaccines as:

$$\begin{array}{l} w_{2}(q_{1},{\;q}_{2})=\sum_{n_{1}=0}^{q_{1}}\sum_{n_{2}=0}^{q_{2}}\;(q_{2,j}-n_{2})p_{1}(n_{1})\;p_{2}(n_{2})\\ +\sum_{n_{1}=q_{1}}^{\infty }\sum_{n_{2}=0}^{q_{2}-(n_{1}-q_{1,j})}\;(q_{2}-n_{2}-(n_{1}-q_{1}))p_{1}(n_{1})\;p_{2}(n_{2}). \end{array}$$ (12)

## Results

We compared each policy performance based on the reductions in unavailability and oversupply by dividing the expected value of baseline policy (P1) by each expected result of new policies (P2), (P3), and (P4), and then subtracting by one. The reduction percentage is to measure the potential cost saving when the new policy is implemented. A higher value indicates a more effective policy in terms of averting vaccine unavailability and unused. To examine the effect of vaccine production, we investigated various scenarios of vaccine supply equal to average demands (regular vaccine supply), vaccine supply quantity that is 25% greater than average demands (excessive vaccine supply), and vaccine supply quantity that is 25% less than average demands (insufficient vaccine supply).

### Regular vaccine supply

**Fig 3:**
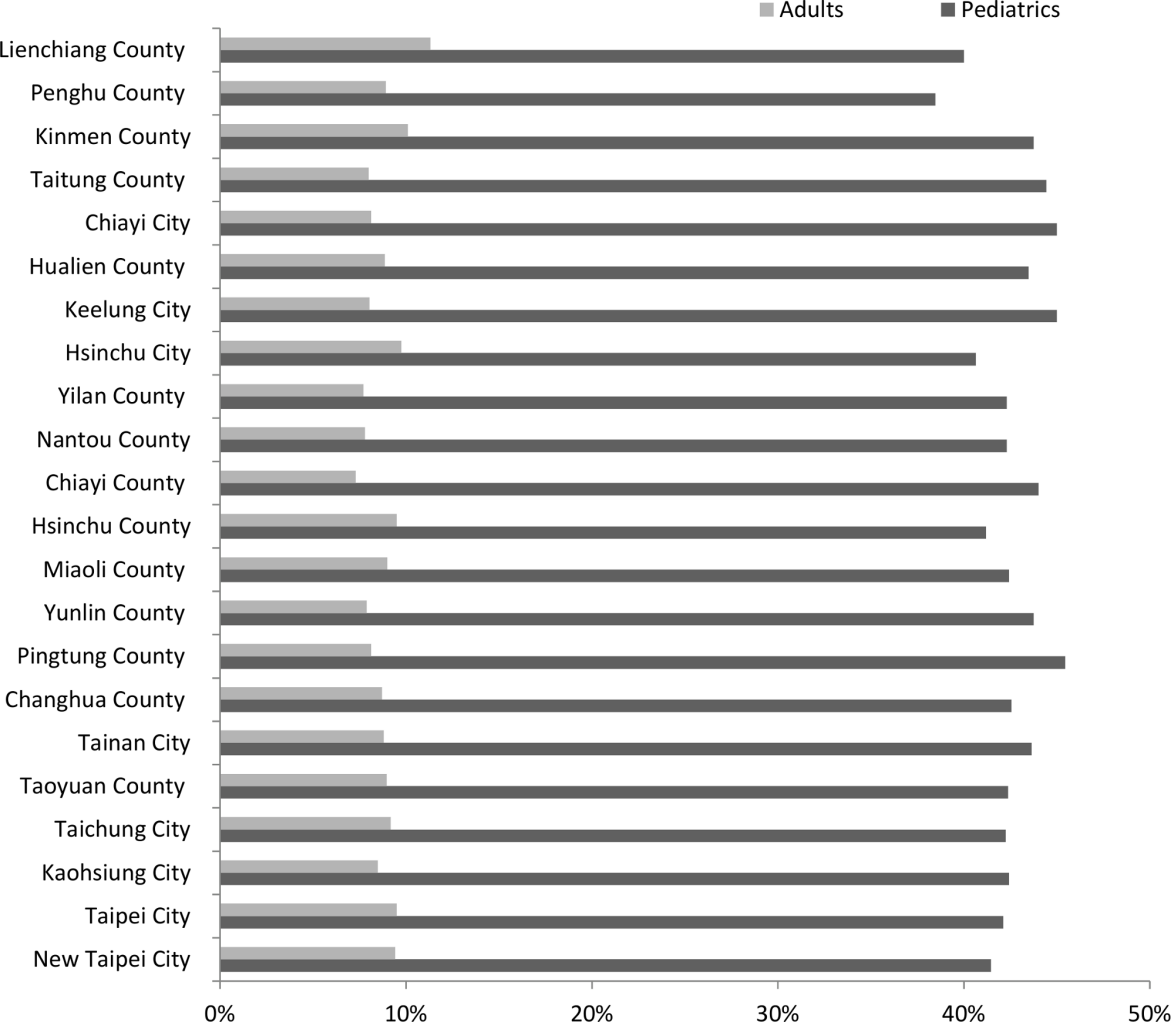
Reduction percentages of vaccine unavailability in the regular vaccine supply scenario.

Fig 3 displayed the cost reduction percentage of vaccine unavailability at each county. Under the sharing policy, the unavailability for pediatrics was reduced by approximately 40% in most counties, whereas the top three counties (Pingtung county, Chiayi city, and Keelung city) obtained more than 45% reduction. The unavailability for adults was decreased approximately 10%. The Lienchiang and Kinmen counties yielded the most reductions of 11.30% and 10.09%, respectively.

**Fig 4:**
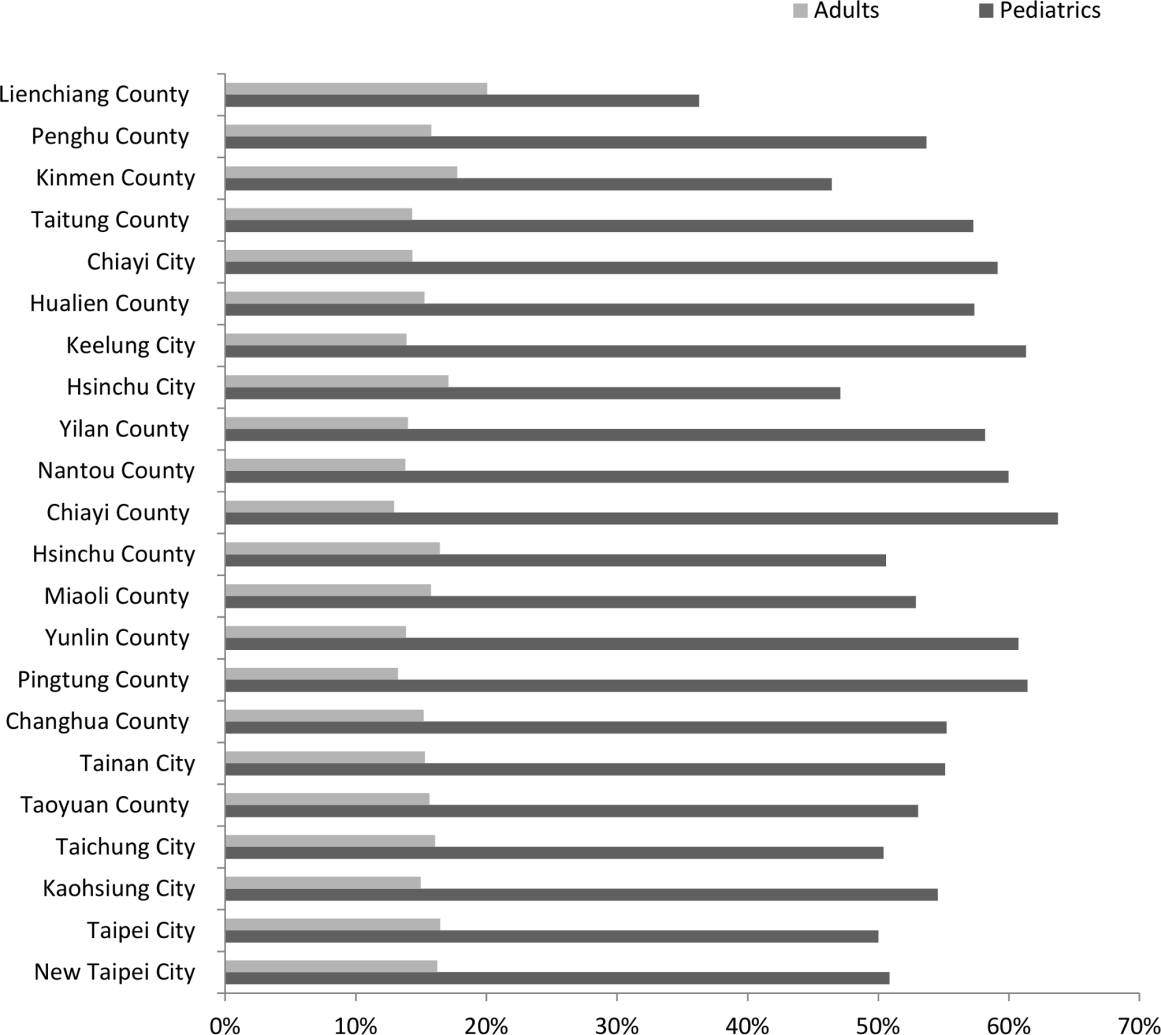
Reduction percentages of vaccine overstock in the regular vaccine supply scenario.

The vaccine-sharing policy reduced the overstock of pediatric vaccines. Similarly, a considerable decrease occurred for both pediatric and adult vaccines. A comprehensive result noted in Fig 4 demonstrates that the expected overstock of pediatric vaccines decreased by more than 50%, and the expected adult vaccine overstock decreased by between 13% and 20% in each location.

Next, we analyze the benefits of redistributing vaccines and the combination of sharing and redistribution vaccines. Table 2 summarizes the reductions of unavailability and overstock of these polices in nationwide. Only sharing vaccine products (P2 policy) reduced the pediatric vaccine unavailability by 43%, adult vaccine unavailability by 9%, the pediatric vaccine overstocks by 54%, and the adult vaccine overstocks by 15%. Only redistributing vaccines (P3 policy) decreased 75% for both unavailability and overstock of pediatric vaccines, and 76% for both unavailability and overstock of adult vaccines. Reduction percentages under the P4 policy spanned 78% to 89% (86% for pediatric vaccine unavailability, 78% for adult vaccine unavailability, 89% for pediatric vaccine overstocks, and 80% for adult vaccine overstocks). As expected, the most effective policy was a combination of sharing and redistributing policies.

**Table 2:** The nationwide reductions in vaccine unavailability and overstock compared to baseline policy under regular vaccine supply.

Policy	Pediatric vaccine unavailability	Adult vaccine unavailability	Pediatric vaccine overstock	Adult vaccine overstock
P1 (baseline)	-	-	-	-
P2	43%	9%	54%	15%
P3	75%	76%	75%	76%
P4	86%	78%	89%	80%

#### Excessive vaccine supply

**Fig 5:**
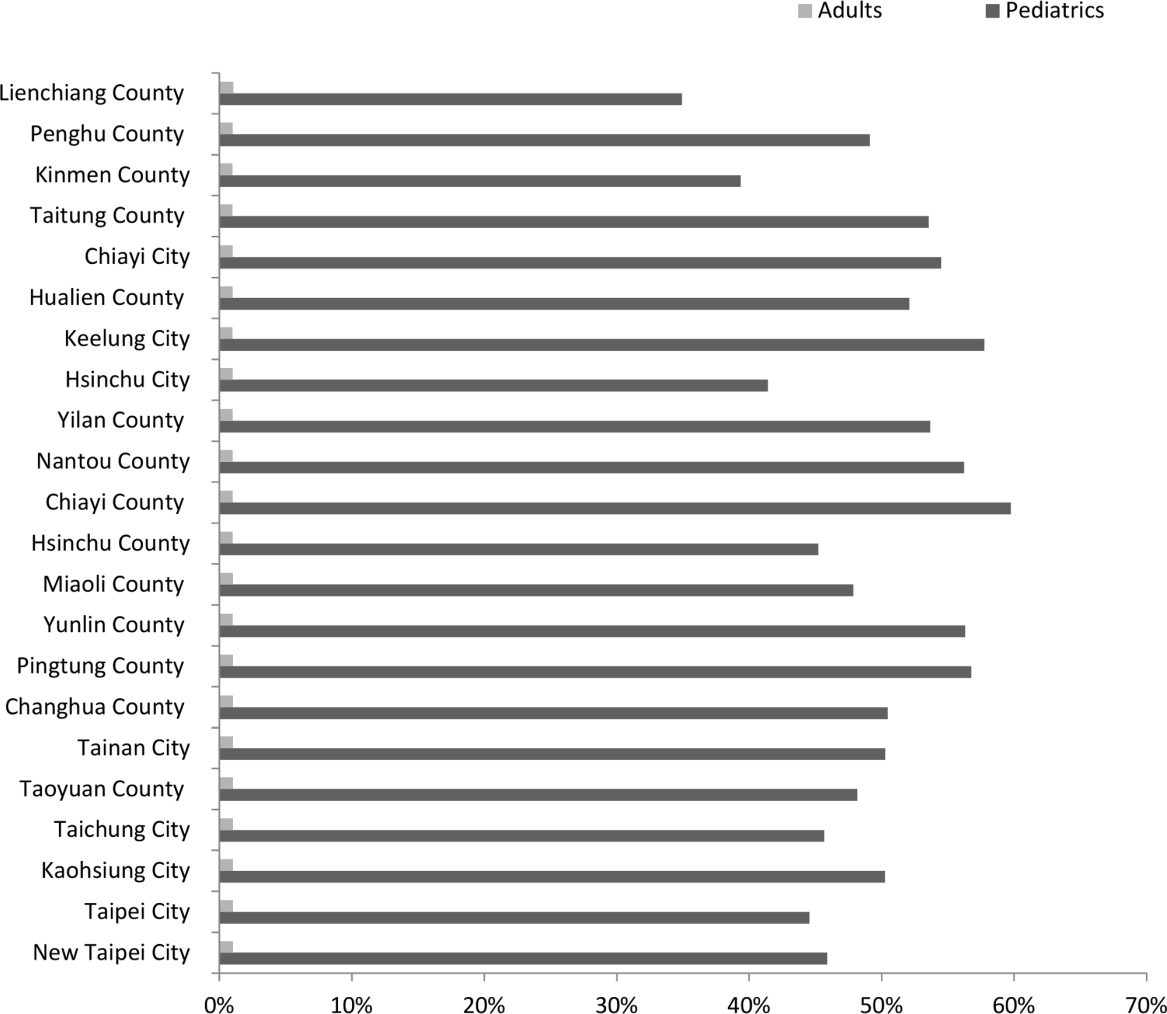
Reduction percentages of vaccine overstock in the excessive vaccine supply scenario.

In this scenario, each county received vaccines of 125% of average demand, and the extra supply ensured that vaccines were available for both pediatrics and adults at vaccination locations. No unavailability was occurred across all polices. In contrast, there was a plenty of vaccine leftovers. Fig 5 reported the reduction percentage by sharing vaccine products, whereas the pediatric vaccine reduced between 35% and 60% across the country. Reductions for adult vaccines were not as significant as the other product with approximately 1% at most locations. The explanation is that most pediatrics are fulfilled, and only few unmet demand would require to access the substituted vaccine.

The overall reduction percentages are summarized in Table 3. There is no unavailability across the four policies, and therefore the reduction percentages of sharing and redistributing vaccines were not reported. This surplus has caused a vast of unused vaccines in the baseline policy. When sharing vaccines, there was a substantial overstock reduction of pediatric vaccine across all countries (by 48%), while the policy only reduced 1% for the adult vaccines. Redistributing vaccine (P3 policy) did not affect to the overstock performance, where the percentages were approximated zero for both pediatric and adult vaccines. The combination of sharing and redistributing (P4 policy) obtained a similar result with the P2 policy. This indicates that in the excessive supply scenario, only sharing vaccine products can reduce unused vaccines.

**Table 3:** The nationwide reduction in vaccine overstock compared to baseline policy under excessive vaccine supply.

Policy	Pediatric vaccine unavailability	Adult vaccine unavailability	Pediatric vaccine overstock	Adult vaccine overstock
P1 (baseline)	-	-	-	-
P2	0%	0%	48%	1%
P3	0%	0%	0%	0%
P4	0%	0%	49%	1%

#### Insufficient vaccine supply

**Fig 6:**
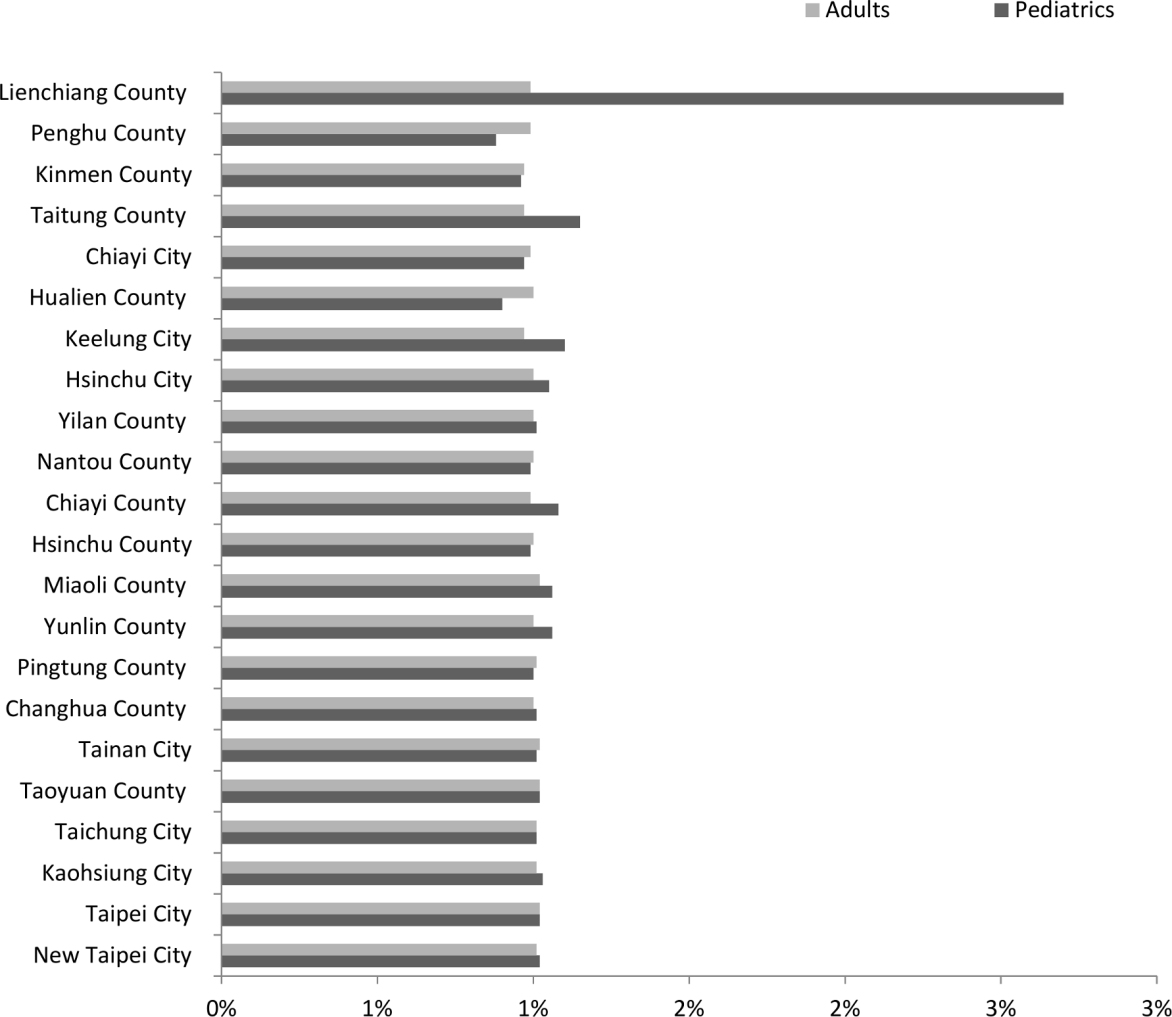
Reduction percentages of vaccine unavailability in the insufficient vaccine supply scenario.

In this scenario, sharing vaccine had insignificant effect on reducing vaccine unavailability. Fig 6 displayed the comparisons of various policies, where reductions for both pediatric and adult vaccines were about 1% in most counties. It is noteworthy that Lienchiang county had the greatest reduction in unavailability of pediatric vaccines among the 22 locations (by 2.7%). For the adult group, sharing vaccines reduced unavailability between 0.97% and 1.02% in most counties.

Table 4 reported the overall reduction in the insufficient ordering scenario. There was no overstock occurred, and therefore we only reported reduction percentages in vaccine unavailability for P2, P3, and P4 policies. The P2 policy decreased by approximately 1% for both pediatric and adult vaccines, and the P3 policy reduced almost zero percent for both vaccine types. The combination policy, P4, cut approximately the same percentage as P2.

**Table 4:** The nationwide reduction in vaccine unavailability compared to baseline policy under insufficient vaccine supply.

Policy	Pediatric vaccine unavailability	Adult vaccine unavailability	Pediatric vaccine overstock	Adult vaccine overstock
P1 (baseline)	-	-	-	-
P2	1%	1%	0%	0%
P3	0%	0%	0%	0%
P4	1%	1%	0%	0%

## Discussion

Implementing either the sharing, redistributing, or a combination of these policies in the existing immunization program can substantially increase vaccine availabilities and reduce vaccine waste. These policies allow more individuals to access vaccines provided by the government. They also mitigate the effect of demand uncertainty to the overall performance.

Vaccine supplies may be excessive or insufficient; such variations must be considered when deciding as to whether to substitute or redistribute vaccines to satisfy different demand groups. To examine these questions, we analyze three supply scenarios: (i) regular vaccine supply; (ii) excessive vaccine supply; and (iii) insufficient vaccine supply. When vaccine supply was as the expected demand, the redistributing policy experienced greater reductions in both unavailability and overstock than the sharing policy. However, the sharing policy performed better than the redistributing policy in other scenarios. No evidence indicated whether the sharing or redistributing policies dominated in all cases. As one may expect, combining the sharing and redistributing policies obtained the best result.

There were distinct performances in sharing pediatric and adult vaccines, and this was impacted by different dose usages when using substitution vaccines. From the overstock aspect, the pediatric vaccine outperformed than the adult vaccine in all supply scenarios (pediatric 54% > adult 15% under regular vaccine supply; pediatric 48% > adult 1% under excessive vaccine supply; no overstock under insufficient vaccine supply). Because each adult demand requires two doses of pediatric vaccine, there is a higher chance to consume the leftover pediatric vaccine. In contrast, each child requires a half-dose of adult vaccine (he or she would actually consume a full dose of adult vaccine due to the safety considerations in substituting adult vaccines for children). From the unavailability aspect, pediatric demands in sharing circumstances obtained a greater reduction than adult demands (pediatric 43% > adult 9% under regular vaccine supply; pediatric 1% ≥ adult 1% under insufficient vaccine supply; no unavailability under excessive vaccine supply). This is due to two factors: (i) the population size of adults is much greater than pediatrics, and thus only a small portion of unfulfilled adult demand can be covered by the surplus pediatric vaccines; and (ii) the adult demand requires double doses than the pediatric demand. In the presence of demand uncertainties, the sharing policy is more beneficial to pediatrics than adults.

This study developed stochastic inventory models to assess the benefit of government’s new policies. We have shown that the expected reductions in overstock and unavailability based on Taiwanese population data and vaccine-substituting protocol information. Our general model can easily adapt to other countries’ data to identify potential synergies across multiple demand sources and vaccine products.

Vaccine ordering decision has direct effects on the implementation of sharing policy. Currently, the Taiwanese government only recommends sharing leftover adult vaccines for unmet pediatric demands. A plausible explanation for the one-way sharing policy is that the pediatric demand might be underestimated when making ordering decision. As a result, there is no chance of administering the unmet adult demands by pediatric vaccines. In this study, we analyze how differences in vaccine ordering quantity can lead to significantly different performances in the sharing policies. Our results indicate that insufficient ordering quantities weaken the performances of sharing polices. This suggests that implementing an immunization programs should consider both sharing and ordering decisions simultaneously. An interesting but still unanswered question is what would be the optimal ordering quantities if both vaccine types can be shared?

This study’s limitations include the vaccination rate data compiled from the previous season, which may not always match the current situation. Additionally, demands between different age groups can be correlated. The assumption of independent demands can be relaxed by the construction of joint probability density function.

Our work embedded with a comprehensive vaccination protocol to assess the economic impact, which may provide insights for the preparation of national immunization program. Despite sharing and redistributing vaccines can improve inventory utilization, implementing such policies requires additional operations and their associated difficulties should be considered in practice. There are potential directions to address these challenges that can enrich the findings of this research. In additional to inventory performances, one may consider vaccination costs including syringes for reconstituting vaccines and extra operations on vaccination. Furthermore, sharing vaccines may increase the risk of vaccination errors. For example, a half-vial of adult vaccine shared among children may cause inaccurate dose administration. Finally, resources may be required for redistributing vaccines, such as transporters, storage spaces and labors. These additional costs would reduce the economic benefits for the new policies. Future study may extend our methods when dealing with these considerations.

## Supporting information

S1 File Supporting information PLOS ONE.xlsx. (XLSX)

## References

[1] Centers for Disease Control, Taiwan. Taiwan National Infectious Disease Statistics System; 2015. Available at: http://nidss.cdc.gov.tw/ch/Default.aspx?op=2 [accessed 07.01.17].

[2] Taiwan Influenza Express Week 20. 2013. Centers for Disease Control, Taiwan; 2014. Available at http://www.cdc.gov.tw/english/list.aspx?treeid=00ED75D6C887BB27&nowtreeid=A49EE318FA9048CE [accessed 07.01.17].

[3] Nichol KL. The efficacy, effectiveness and cost-effectiveness of inactivated influenza virus vaccines. Vaccine 2003; 21(16):1769–1775. 12686092 10.1016/s0264-410x(03)00070-7

[4] Nichol KL. Efficacy and effectiveness of influenza vaccination. Vaccine 2008; 26(4): 17–22. 10.1016/j.vaccine.2008.07.048 19230153

[5] Esposito S, et al Clinical and economic impact of influenza vaccination on healthy children aged 2–5 years. Vaccine 2006; 24(5): 629–635. doi: 10.1016/j.vaccine.2005.08.054 16157429 10.1016/j.vaccine.2005.08.054

[6] Meltzer MI, Neuzil KM, Griffin MR and Fukuda K. An economic analysis of annual influenza vaccination of children. Vaccine 2005; 23(8): 1004–1014. doi: 10.1016/j.vaccine.2004.07.040 15620473 10.1016/j.vaccine.2004.07.040

[7] Mullooly JP, et al Influenza vaccination programs for elderly persons: Cost-effectiveness in a health maintenance organization. Annals of Internal Medicine 1994; 121(12): 947–952. 7978721 10.7326/0003-4819-121-12-199412150-00008

[8] Chen CH, Chiu PJ, Chih YC, Yeh GL. Determinants of influenza vaccination among young Taiwanese children, Vaccine 2015; 33(16): 1993–1998. doi: 10.1016/j.vaccine.2015.01.032 25613722 10.1016/j.vaccine.2015.01.032

[9] Chiu PJ, Chen CH, Chih YC. Effectiveness of the influenza vaccination program for the elderly in Taiwan, Vaccine 2013; 31(4): 632–638. doi: 10.1016/j.vaccine.2012.11.055 23212027 10.1016/j.vaccine.2012.11.055

[10] Wang ST, Lee LT, Chen LS, Chen TH. Economic evaluation of vaccination against influenza in the elderly: an experience from a population-based influenza vaccination program in Taiwan. Vaccine 2005; 23(16):1973–1980. doi: 10.1016/j.vaccine.2004.10.011 15734070 10.1016/j.vaccine.2004.10.011

[11] Wang CS, Wang ST, Lai CT, Lin LJ, Chou P. Impact of influenza vaccination on major cause-specific mortality. Vaccine 2007; 25(7):1196–1203. doi: 10.1016/j.vaccine.2006.10.015 17097773 10.1016/j.vaccine.2006.10.015

[12] Wang CS, Wang ST, Lai CT, Lin LJ, Lee CT, Chou P. Reducing major cause-specific hospitalization rates and shortening hospital stays after influenza vaccination. Clinical Infectious Diseases 2004, 39(11):1604–1610. 10.1086/425323 15578359

[13] Maciosek MV, Solberg LI, Coffield AB, Edwards NM, Goodman MJ. Influenza vaccination: Health impact and cost effectiveness among adults aged 50 to 64 and 65 and older. Am J Prev Med 2006; 31: 72–9. doi: 10.1016/j.amepre.2006.03.008 16777545 10.1016/j.amepre.2006.03.008

[14] Turner DA, Wailoo AJ, Cooper NJ, Sutton AJ, Abrams KR, Nicholson KG. The cost-effectiveness of influenza vaccination of healthy adults 50–64 years of age. Vaccine 2006; 24(7): 1035–1043. doi: 10.1016/j.vaccine.2004.12.033 16183177 10.1016/j.vaccine.2004.12.033

[15] Nichol KL, Nordin JD, Nelson DB, Mullooly JP, Hak E. Effectiveness of influenza vaccine in the community-dwelling elderly. New England Journal of Medicine 2007; 357: 1373–1381. doi: 10.1056/NEJMoa070844 17914038 10.1056/NEJMoa070844

[16] Chen SC, Liao CM. Cost-effectiveness of influenza control measures: a dynamic transmission model-based analysis. Epidemiology & Infection. 2013, 141(12):2581–2594. 23481024 10.1017/S0950268813000423 PMC9151383

[17] Centers for Disease Control, Taiwan. Press Releases; 2013. Available at: http://www.cdc.gov.tw/info.aspx?treeid=45da8e73a81d495d&nowtreeid=1bd193ed6dabaee6&tid=68E5FF1AD30300D1 [accessed 07.01.17].

[18] Centers for Disease Control, Taiwan. Influenza Vaccine Vaccination Plan; 2014. Available at: http://www.cdc.gov.tw/professional/page.aspx?treeid=BEAC9C103DF952C4&nowtreeid=5D8AD59FB1140E86 [accessed 07.01.17].

[19] Centers for Disease Control, Taiwan. Press Releases, 23 September 2014. Available at: http://www.cdc.gov.tw/professional/info.aspx?treeid=cf7f90dcbcd5718d&nowtreeid=f94e6af8daa9fc01&tid=FCE41B569F00497A [accessed 07.01.17].

[20] Centers for Disease Control, Taiwan; 2012. Available at: http://www.cdc.gov.tw/professional/info.aspx?treeid=beac9c103df952c4&nowtreeid=a59c545d09b7af24&tid=D0FB14AD87E98DBB [accessed 07.01.17].

[21] Chang YC, Huang N, Chen LS, Hsu SW, Chou YJ. Factors affecting repeated influenza vaccination among older people in Taiwan. Vaccine 2013, 31(2): 410–416. doi: 10.1016/j.vaccine.2012.10.086 23142305 10.1016/j.vaccine.2012.10.086

[22] Chang YC, Tung HJ, Hsu SW, Chen LS, Kung PT, Huang KH, et al Use of seasonal influenza vaccination and its associated factors among elderly people with disabilities in Taiwan: A population-based study. PLoS ONE 2016; 11(6):e0158075 doi: 10.1371/journal.pone.0158075 27336627 10.1371/journal.pone.0158075 PMC4919006

[23] Plans-Rubió P. The vaccination coverage required to establish herd immunity against influenza viruses. Preventive Medicine 2012; 55(1): 72–77. doi: 10.1016/j.ypmed.2012.02.015 22414740 10.1016/j.ypmed.2012.02.015

[24] Lu CM, Chang CM, Kao CC and Chi YC. The benefits of influenza vaccine information network construction. 2008 Joint Conference on Medical Information in Taiwan 2008; 65–69.

[25] Centers for Disease Control, Taiwan. Lists of Contracted Hospitals and Clinics for Disease Prevention and Vaccinations; 2014.Available at: http://www.cdc.gov.tw/vacunit.aspx?treeid=d78de698c2e70a89&nowtreeid=a7af893fd64d1aab [accessed 07.01.17].

[26] Centers for Disease Control, Taiwan. Press Releases, 23 December 2014. Available at: http://www.cdc.gov.tw/professional/info.aspx?treeid=cf7f90dcbcd5718d&nowtreeid=f94e6af8daa9fc01&tid=7B8DA7ECE150E5F0 [accessed 07.01.17].

[27] Centers for Disease Control and Prevention. Seasonal Influenza Vaccine Dosage and Administration; 2015. Available at: http://www.cdc.gov/flu/about/qa/vaxadmin.htm [accessed 07.01.17].

[28] Department of Statistics, Ministry of the Interior, Taiwan. Monthly Bulletin of Interior Statistics; 2014. Available at: http://sowf.moi.gov.tw/stat/month/list.htm [accessed 07.01.17].

[29] Lee CL, Chen TY, Chi YC, Chou SM, Chen CS and Yang CH. The overview of government-funded influenza vaccination program during influenza season 2011–2012. Taiwan Epidemiology Bulletin 2013; 29(20): 253–267.

